# Infiltrated IL-17A-producing gamma delta T cells play a protective role in sepsis-induced liver injury and are regulated by CCR6 and gut commensal microbes

**DOI:** 10.3389/fcimb.2023.1149506

**Published:** 2023-07-05

**Authors:** Jian Wan, Qian Zhang, Yilong Hao, Zhang Tao, Wei Song, Song Chen, Long Qin, Weidong Song, Yi Shan

**Affiliations:** ^1^ Department of Emergency and Critical Care Medicine, Shanghai Pudong New Area People’s Hospital, Shanghai, China; ^2^ Department of Emergency and Critical Care Medicine, Second Affiliated Hospital of Naval Medical University, Shanghai, China

**Keywords:** IL-17A, gamma delta T cells, CCR6, microbiota, sepsis, liver injury

## Abstract

**Introduction:**

Sepsis is a common but serious disease in intensive care units, which may induce multiple organ dysfunctions such as liver injury. Previous studies have demonstrated that gamma delta (γδ) T cells play a protective role in sepsis. However, the function and mechanism of γδ T cells in sepsis-induced liver injury have not been fully elucidated. IL-17A-producing γδ T cells are a newly identified cell subtype.

**Methods:**

We utilized IL-17A-deficient mice to investigate the role of IL-17A-producing γδ T cells in sepsis using the cecum ligation and puncture (CLP) model.

**Results:**

Our findings suggested that these cells were the major source of IL-17A and protected against sepsis-induced liver injury. Flow cytometry analysis revealed that these γδ T cells expressed Vγ4 TCR and migrated into liver from peripheral post CLP, in a CCR6-dependent manner. When CLP mice were treated with anti-CCR6 antibody to block CCR6-CCL20 axis, the recruitment of Vγ4+ γδ T cells was abolished, indicating a CCR6-dependent manner of migration. Interestingly, pseudo germ-free CLP mice treated with antibiotics showed that hepatic IL-17A+ γδ T cells were regulated by gut commensal microbes. *E. coli* alone were able to restore the protective effect in pseudo germ-free mice by rescuing hepatic IL-17A+ γδ T cell population.

**Conclusion:**

Our research has shown that Vγ4+ IL-17A+ γδ T cells infiltrating into the liver play a crucial role in protecting against sepsis-induced liver injury. This protection was contingent upon the recruitment of CCR6 and regulated by gut commensal microbes.

## Introduction

Sepsis is an uncontrolled inflammation caused by infection with organ dysfunction (Cheng et al., 2017). More than 1.5 million patients with sepsis are annually admitted to the intensive care unit (ICU) in the US, leading to 30% mortality annually (Seymour et al., 2016). Although a lot of progress in the pathology of the disease has been made, the role of sepsis in multiple organ failure remains elusive. The liver plays a crucial role in the pathology of sepsis (Strnad et al., 2017), and sepsis-related liver injury is associated with high mortality (Baggio Savio et al., 2017). Therefore, it is necessary to explore the pathogenesis of sepsis-induced liver injury and to develop effective therapies for this disease.

Gamma delta (γδ) T cells are enriched in the liver, with a frequency of 3%–5% of the total liver lymphocytes (Racanelli and Rehermann, 2006). These cells bridge the innate and adaptive immune responses and secrete pro-inflammatory cytokines, including interleukin 17A (IL-17A) and interferon gamma (IFN-γ), upon stimulation (Bonneville et al., 2010). IL-17A-producing γδ T cells can be considered as a separate cell type, and they play a crucial role in host defense against infections such as bacteria, fungi, and viruses (Hayday, 2009). Moreover, IL-17A^+^ γδ T cells have been identified as playing a protective role in polymicrobial sepsis (Ogiku et al., 2012), particularly in organ failure of the lung (de Souza Costa et al., 2015). Although it has been reported that γδ T cells are involved in sepsis-related liver injury, their physiological characteristics are largely unclarified.

The thymus is the primary organ for γδ T-cell maturation and differentiation. Unlike conventional alpha beta (αβ) T cells, the majority of γδ T cells acquire their effector function in the thymus. These cells predominantly reside in the mucosal and epithelial barriers and produce cytokines rapidly, although a small fraction still circulate in the periphery (Chien et al., 2014; Nielsen et al., 2017; Ma et al., 2019). According to their function potential and localization, γδ T cells can be further divided into effector subsets with bias on the use of T-cell receptor (TCR) gene segments. However, the role of the different γδ T-cell subtypes in sepsis has not been clarified. The origin of these γδ T cells is also unclear.

CCR6 is an important receptor in mucosal immunity, with CCL20 as the chemokine ligand (Cook et al., 2000). Among the CD4 T cells, CCR6 is specifically expressed on T helper 17 (Th17) cells and regulatory T cells (Tregs). It can mediate distinct immune diseases through the chemotaxis of T cells. Recently, researchers have identified that CCR6 is also expressed in γδ T cells, functionally associated with subsets of IL-17A production (Oo et al., 2012). However, the function of CCR6 expressed in γδ T cells in liver disease is largely unclear.

In the past decade, the gut microbiota was considered as a major modulator of the injury responses in organs remote from the gastrointestinal tract, including the liver (Llorente and Schnabl, 2015; Schuijt et al., 2016; Hu et al., 2017; Li et al., 2018; Tripathi et al., 2018). It has also been reported that dysbiosis could increase the risk of sepsis (Liu et al., 2019) and death (Salio et al., 2014). Germ-free (GF) mice with cecum ligation and puncture (CLP) showed higher pathogen load and mortality (Luoma et al., 2014). The gut microbiota has been shown to be involved in sepsis progression (Gong et al., 2019) and reported to regulate the host immune responses in disease pathogenesis (Chassaing et al., 2014). Thus, the gut microbiota could be an important mediator of sepsis-related organ failure, which still needs to be further elucidated.

In this study, Vγ4^+^ γδ T cells were revealed to play a protective role in sepsis-induced liver injury through the production of IL-17A. The CCR6–CCL20 axis was investigated in sepsis-induced liver injury, which demonstrated a CCR6-dependent manner of recruitment of γδ T cells into the liver. The gut microbiota was also revealed to be positively correlated with sepsis-induced liver injury.

## Materials and method

### Mice

C57BL/6 mice were purchased from Shanghai SLAC Laboratory Animal Co., Ltd. (Shanghai, China). IL-17A knockout (KO), TCRαβ^−/−^, TCRγδ^−/−^, CD45.1, and *Rag1* mice were obtained from the Model Animal Research Center (Nanjing University, Nanjing, China). All mice were housed in a specific pathogen-free facility at the National Center for Protein Science Shanghai. All the mice in this study were males, 8–12 weeks old.

### Ethical statement

All animal protocols were approved by the Ethics Committee for Animal Care and Use at Shanghai Pudong New Area People’s Hospital.

### Antibody treatment

The anti-mouse Vγ4 antibody (eBioscience, San Diego, CA, USA) was intraperitoneally (i.p.) administered at 500 μg, 3 days before CLP (de Souza Costa et al., 2015). Blockade of the CCR6^+^ population was conducted through i.p. administration of an anti-mouse CCR6 antibody (BioLegend, San Diego, CA, USA) at 500 μg, 3 days before CLP (Hammerich et al., 2014), which could block the function of the CCR6–CCL20 axis by binding CCR6 on the cell surface. Control animals received an i.p. injection of isotype immunoglobulin G (IgG; 500 μg, 3 days before CLP) (BioLegend).

### Sepsis by CLP

Sepsis was induced by CLP (Rittirsch et al., 2009). Briefly, C57BL/6 mice were anesthetized with Avertin (300 mg/kg, i.p) and a 1-cm midline incision made on the anterior abdomen. The cecum was exposed and ligated below the ileocecal junction without causing bowel obstruction. Puncture was performed with an 18-gauge needle five times to induce sepsis. The cecum was squeezed to allow the contents to pass through the punctures. The cecum was then placed back into the abdominal cavity and the peritoneal wall and skin incision closed. All animals were resuscitated with 1 m of serial saline subcutaneously.

### Survival analysis

After surgery, the mice were monitored for survival status every 24 h for 7 days. The overall survival analysis was performed using the Mantel–Cox test with Prism v.5 software.

### Bacterial load assessment

Serum samples were serially diluted with sterile phosphate-buffered saline (PBS) and cultured on agar plates. After incubation at 37°C overnight, the colony was counted. According to the dilution of the samples, the limit detection for the colony-forming unit (CFU) assay was 1 × 10^4^ CFU/ml.

### Cell preparation

The mice were euthanized and the thymus, spleen, and liver removed for cell collection. For the thymus, the tissue was ground and passed through a 70-μm mesh to obtain single-cell suspension. The spleen was first ground and passed through a 70-μm mesh, and then red blood cells (RBCs) were lysed and a single-cell suspension in PBS obtained. Similarly, the liver was ground and passed through a 70-μm mesh. The cell suspension was resuspended in 40% Percoll (GE Healthcare, Chicago, IL, USA) and centrifuged for 20 min at 2,800 rpm with breaking at 1. Cells were then collected from precipitates and washed in PBS twice.

### γδ T-cell isolation

Splenic or thymic single-cell suspensions were processed through immune magnetic negative selection to deplete αβ T cells, B220^+^ cells, NK1.1^+^ cells, and MHCII^+^ cells, then positively selected using phycoerythrin (PE)-conjugated anti-TCRγδ (BioLegend) and anti-PE microbeads (Miltenyi Biotec, Bergisch Gladbach, Germany).

### Flow cytometry analysis

The cell suspensions were blocked with anti-mouse CD16/32 (BioLegend) at 1:200 dilution and stained with surface markers including anti-mouse CD3, TCRβ, TCRγδ, TCRVγ4, TCRVγ6, and CCR6 for 30 min at 4°C.

For cytokine staining, the cell suspensions were stimulated with phorbol 12-myristate 13-acetate (PMA; 50 ng/ml), ionomycin (500 ng/ml) (both from Sigma, St. Louis, MO, USA), and GolgiPlug (BD Biosciences, Franklin Lakes, NJ, USA) for 4 h at 37°C in an incubator. After surface marker staining, the cells were fixed and permeabilized (BD Biosciences) and stained for cytokine IL-17A or IFN-γ at 4°C for 30 min. The samples were analyzed with Fortessa X-20 (BD Biosciences).

### Adenoviral knockdown

To knock down the expression of CCR6, a specific RNAi vector of CCR6 was designed using RNAi Designer, with the sequence CCGATAACATCAATGTCCAAGTGAA. Subsequently, 293T cells were transfected with this vector to produce adenovirus. The BLOCK-iT vector was used as a control. The γδ T cells were isolated and stimulated with anti-CD3 and anti-CD28 for 24 h. Adenoviral supernatants were then added into the cell culture, and the efficiency of RNAi was evaluated using flow cytometry before adoptive transfer.

### Adoptive transfer

Recipient mice (6–8 weeks old) were sublethally irradiated with 5 Gy a day prior to adoptive transfer and were maintained with antibiotics containing water for 2 weeks. On the second day, 0.1 million γδ T cells from the thymus or the liver were adoptively transferred into recipients by i.p. injection.

### Real-time PCR

RNA was isolated from the liver using the TRIzol reagent (Invitrogen, Carlsbad, CA, USA) according to the manufacturer’s instructions. Complementary DNA (cDNA) was synthesized using reverse transcriptase (Promega, Madison, WI, USA). A real-time PCR kit (Takara, San Jose, CA, USA) was used according to the manufacturer’s instructions. The reaction was performed on an ABI 7500 Real-Time PCR system. The primer sequences used in this work are listed in **Table 1**.

**Table 1:** qPCR primers used in this study.

	Forward (5′–3′)	Reverse (5′–3′)
*β-actin*	GATGGACTCCTCAGATGAGATG	CAAACAGCGTCTGATATCCATG
*Il17a*	GCCTCTTTGGGTCAACTTAATG	GAGGTTTTCTGTTGGACAAGTC
*Ccl20*	TTTCTGTGAGAGTGGTCTTACC	AGTCTGAGAGATGACATTGCAA

### Serum alanine transaminase measurement

The serum alanine transaminase (ALT) levels were measured using ALT assay kits (Nanjing Jiancheng Bioengineering Institute) according to the manufacturer’s instructions. According to the sample dilution, the limit of detection for ALT measurement was 1 U/L.

### Enzyme-linked immunosorbent assay

The IL-17A levels in the serum and liver were determined using IL-17A enzyme-linked immunosorbent assay (ELISA) kits (ThermoFisher, Waltham, MA, USA) according to the manufacturer’s instructions. According to the sample dilution, the limit of detection of the sample was 1.6 pg/ml.

### Depletion of microbiota with broad-spectrum antibiotics

To deplete the gut microbiota and generate pseudo-GF mice, a broad-spectrum antibiotic cocktail (100 mg/kg vancomycin, 200 mg/kg neomycin sulfate, 200 mg/kg metronidazole, and 200 mg/kg ampicillin) was intragastrically administered once a day for 5 days to 8- to 12-week-old mice.

### Fecal microbiota transplantation assay

Fecal microbiota transplantation (FMT) was performed according to a modified method previously described (DeFronzo et al., 2016; Buford, 2017). Briefly, recipient mice were administered the suspension *via* oral gavage once a day for 3 days with 200 μl fecal dilution in PBS. To prepare this dilution, fecal samples were collected from donors. Stool pellets were pooled and vortexed for 5 min in 0.125 g/L PBS for homogenization. Thereafter, the samples were gently centrifuged for 5 min at 350 × *g* and the supernatant collected. Subsequently, 200 μl of this suspension was administered by oral gavage to recipient mice.

### Statistical analysis

Statistical analysis was performed with GraphPad Prism 5.0 (GraphPad Software, La Jolla, CA, USA). Data were presented as the mean ± SEM. Differences between two groups were assessed using Student’s *t*-test, whereas those among more than two groups were determined using ANOVA. A *p*-value <0.05 was considered significant (*p < 0.05, **p < 0.01, ***p < 0.001).

## Results

### IL-17A protected against sepsis-induced liver injury in cecum ligation and puncture mice

**Figure 1:**
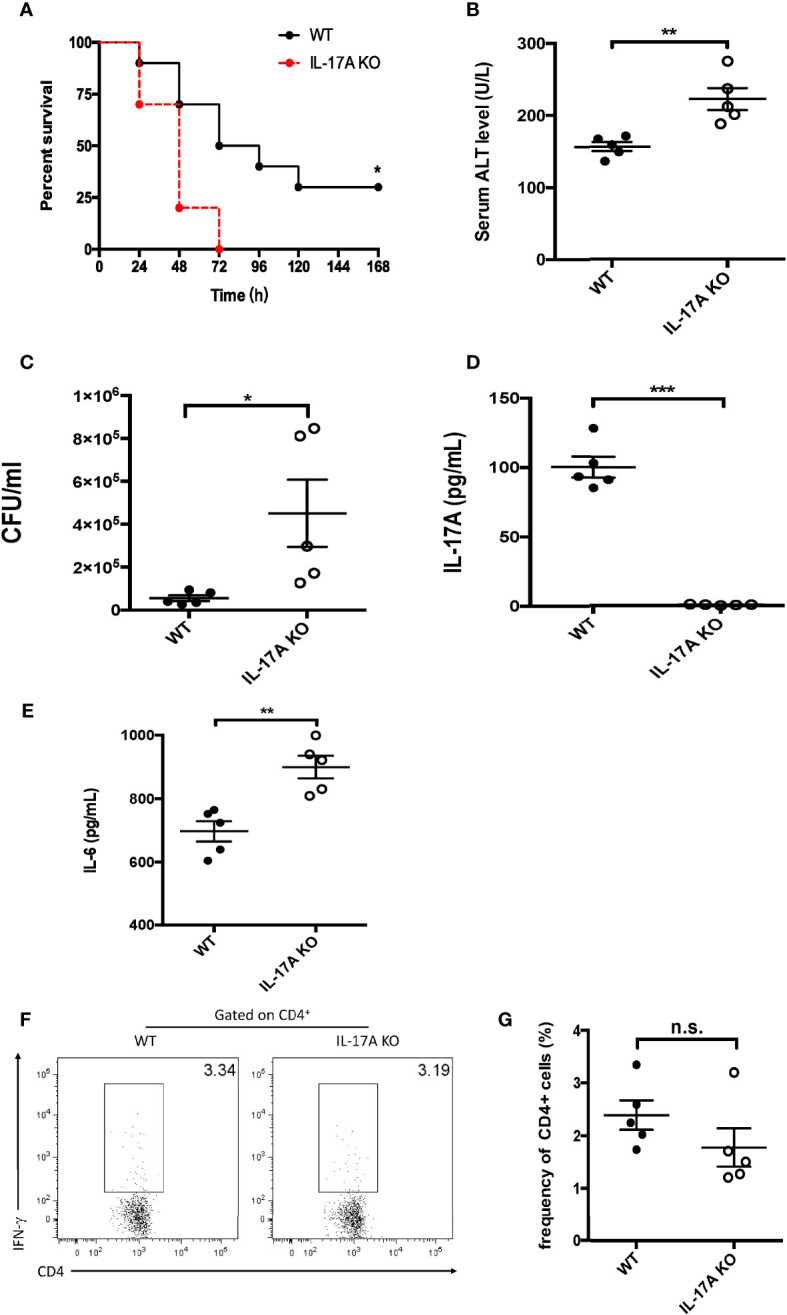
Characteristics of interleukin 17A (IL-17A) knockout (KO) mice after cecum ligation and puncture (CLP). Wild-type (WT) and IL-17A KO mice were subjected to CLP. **(A)** Survival curves of WT and IL-17A KO mice subjected to CLP. **(B)** Serum alanine transaminase (ALT) levels between WT and IL-17A KO mice determined as described post-CLP. **(C)** Bacterial colony-forming units (CFUs) in the blood between WT and IL-17A KO mice subjected to CLP measured using agarose plates. **(D, E)** Serum levels of IL-17A and IL-6 between WT and IL-17A KO mice subjected to CLP determined using ELISA. **(F)** Representative fluorescence-activated cell sorting (FACS) plots of splenic T helper 1 (Th1) cells between WT and IL-17A KO mice subjected to CLP. **(G)** Statistics of Th1 cells in CD4 T cells. Data are from one intact experiment and representative of three independent experiments (five mice per group). *Error bars* denote the mean ± SEM. Survival analysis was performed using Mantel–Cox, while the rest was performed using Student’s *t*-test. *n.s.*, not significant. *p < 0.05, **p < 0.01, ***p < 0.001.

To assess the effects of IL-17A on sepsis-induced liver injury, IL-17A-deficient mice were subjected to sepsis using CLP. As shown in **Figure 1A**, all animals in the IL-17A-deficient group died within 72 h post-CLP. In contrast, 30% of the wild-type (WT) mice survived 7 days post-CLP (**Figure 1A**). The serum level of ALT was increased in the WT group 12 h post-CLP, whereas it was even higher in the IL-17A KO group (**Figure 1B**). In addition, the bacterial load in the blood of IL-17A-deficient mice was significantly higher than that of control animals (**Figure 1C**). As expected, the serum levels of IL-17A were below the detection levels in the deficient group (**Figure 1D**). Moreover, the serum IL-6 level was much higher in the IL-17A KO group than that in the WT group (**Figure 1E**). However, in the spleen, the results revealed little differences in the IFN-γ^+^ CD4 T cells between the control and IL-17A-deficient mice (**Figures 1F, G**). Together, these marked differences between the control and KO groups indicate a protective role of IL-17A in liver injury in CLP-induced sepsis.

### γδ T cells were the major source of IL-17A in sepsis-induced liver injury

**Figure 2:**
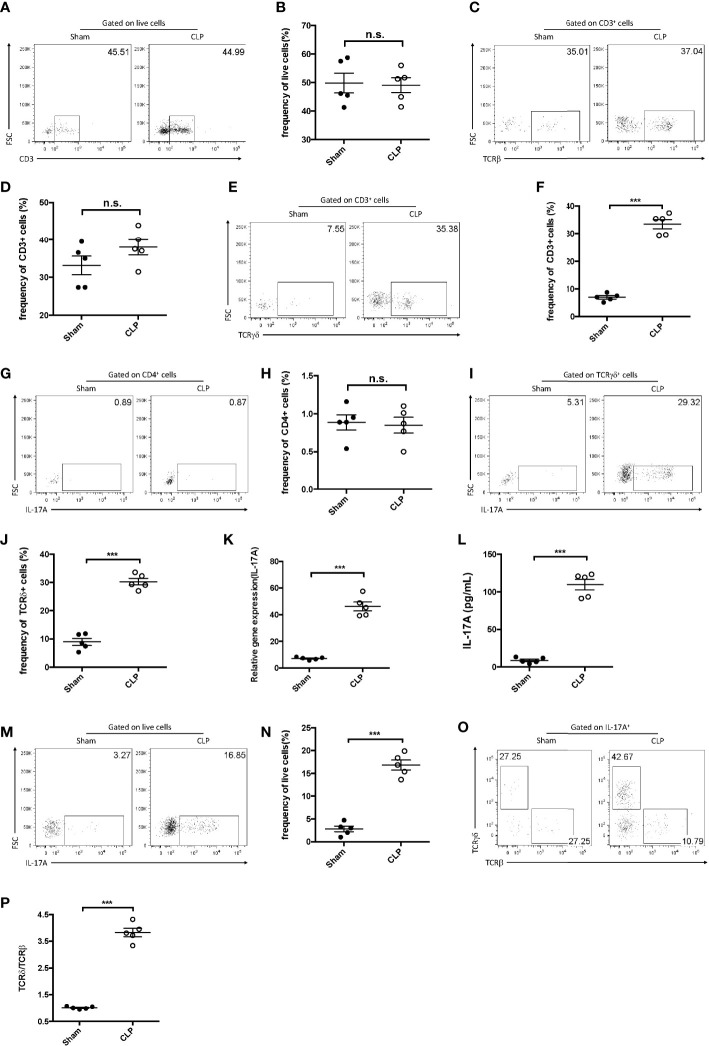
IL-17A^+^ gamma delta (γδ) T cells were enriched in the liver after cecum ligation and puncture (CLP). C57BL/6 mice were subjected to CLP and analyzed 12 h later. **(A, C, E)** Representative fluorescence-activated cell sorting (FACS) plots of CD3^+^ **(A)**, TCRβ^+^ **(C)**, and TCRγδ^+^ **(E)** cells in the liver between sham and CLP mice. **(B, D, F)** Statistics of CD3^+^ **(B)**, TCRβ^+^ **(D)**, and TCRγδ^+^ **(F)** cells in (Cheng et al., 2017). **(G, I)** Representative FACS plots of IL-17A^+^ cells among the CD4^+^ cells **(G)** and the γδ T cells **(I)** in the liver between sham and CLP mice. **(H, J)** Statistics of IL-17A^+^ **(H)** and IL-17A **(J)** cells in (Cheng et al., 2017). **(K)** Transcriptional levels of IL-17A in the liver between sham and CLP mice measured using quantitative PCR (qPCR). **(L)** Serum levels of IL-17A between sham and CLP mice measured using ELISA. **(M)** Representative FACS plots of IL-17A^+^ cells among the total live cells in the liver between sham and CLP mice. **(N)** Statistics of IL-17A^+^ cells in (Cheng et al., 2017). **(O)** Representative FACS plots of TCRβ^+^ and TCRδ^+^ cells among the IL-17A^+^ cells in the liver between sham and CLP mice. **(P)** Statistics of the TCRδ *vs*. TCRβ ratio in (Cheng et al., 2017). Data are from one intact experiment and representative of three independent experiments (five mice per group). *Error bars* denote the mean ± SEM. Statistical analysis was performed using Student’s *t*-test. *n.s.*, not significant. ***p < 0.001.

As multiple populations could secrete IL-17A, its major source was further examined in the liver post-CLP. Single-cell suspensions of the liver were prepared for flow cytometry analysis 12 h after the CLP procedure. More CD3^+^ T lymphocytes were detected in sepsis-induced livers compared to the control group (**Figures 2A, B**). To better determine the subtypes, the expression of αβ and γδ TCRs in these T lymphocytes was analyzed. It was found that, although there was little difference in the expression of αβ TCRs (**Figures 2C, D**), there were more γδ TCR^+^ T lymphocytes in CLP livers than in controls (**Figures 2E, F**). In addition, IL-17A was not detected among the αβ T cells in either the CLP or the control group (**Figures 2G, H**), while 30% of the γδ T cells were determined as IL-17A-producing cells in CLP mice (**Figures 2I, J**). Thus, more IL-17A^+^ γδ TCR cells were detected in the CLP group.

Moreover, hepatic IL-17A levels were significantly higher in CLP livers than in controls, as determined using both quantitative PCR (qPCR) and ELISA (**Figures 2K, L**). The fluorescence-activated cell sorting (FACS) plots showed that IL-17A^+^ cells accounted for approximately 20% of the hepatic live cells from CLP mice (**Figures 2M, N**). To further address which subset produced more IL-17A in CLP livers, the expression of the αβ and γδ TCRs in these cells was examined. It was discovered that more than 40% of the IL-17A^+^ lymphocytes were TCRδ^+^, but only about 10% were TCRβ^+^ in CLP mice (**Figures 2O, P**), indicating that hepatic γδ T cells comprise the major subset for IL-17A secretion in sepsis-induced liver injury.

### IL-17A-producing γδ T cells played a major protective role in sepsis-induced liver injury

**Figure 3:**
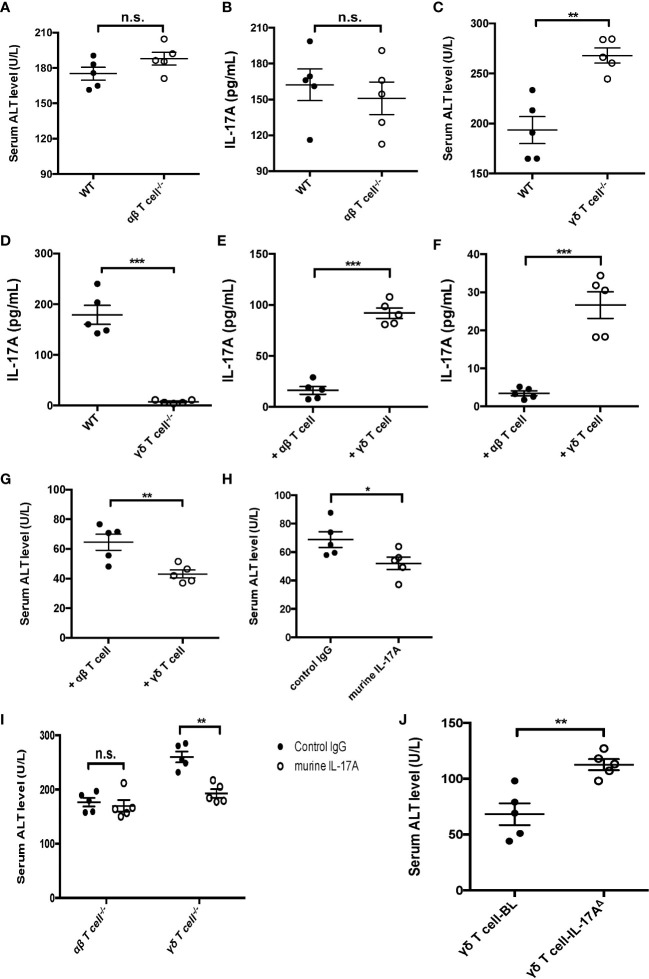
Function of IL-17A^+^ gamma delta (γδ) T cells in cecum ligation and puncture (CLP)-induced liver injury. Wild-type (WT) and alpha beta (αβ) T-cell knockout (KO) mice were subjected to CLP. **(A, B)** Serum levels of alanine transaminase (ALT) measured as described **(A)** and of interleukin 17A (IL-17A) determined using ELISA **(B)**. WT and γδ T-cell KO mice were subjected to CLP. **(C, D)** Serum levels of ALT measured as described **(C)** and of IL-17A determined using ELISA **(D)**. *Rag1* mice were adoptive transferred with αβ or γδ T cells isolated from WT mice and subjected to CLP. **(E, F)** The levels of IL-17A in the plasma (Cheng et al., 2017) and liver (Cheng et al., 2017) were determined using ELISA. **(G)** Serum levels of ALT were measured after adoptive transferring. **(H)** *Rag1* mice treated with control immunoglobulin G (IgG) or murine IL-17A through intraperitoneal injection and subjected to CLP. **(I)** αβ and γδ T-cell KO mice treated with murine IL-17A through intraperitoneal injection of isotype IgG as control and subjected to CLP. **(J)** *Rag1* mice adoptive transferred with BL-vehicle- or BL-IL-17A^Δ^-transfected γδ T cells and then subjected to CLP. The serum levels of ALT were measured as described. Data are from one intact experiment and representative of three independent experiments (five mice per group). *Error bars* denote the mean ± SEM. Statistical analysis was performed using Student’s *t*-test. *n.s.*, not significant. *p < 0.05, **p < 0.01, ***p < 0.001.

To better clarify the function of γδ T cells expressing IL-17A in sepsis-induced liver injury, CLP was performed on αβ and γδ TCR KO mice, which were deficient in αβ and γδ T cells, respectively. The data showed that there was little difference in the serum ALT and IL-17A between the WT and αβ T-cell KO mice at 12 h post-CLP (**Figures 3A, B**). However, the knockout of γδ T cells led to a markedly elevated plasma ALT but a reduced IL-17A level, compared to WT littermates (**Figures 3C, D**). On the other hand, adoptive transfer of γδ T cells, but not αβ T cells, could rescue the levels of IL-17A in both serum and liver (**Figures 3E, F**) and suppress plasma ALT (**Figure 3G**). Similar results were observed in *Rag1* mice with exogenous IL-17A (**Figure 3H**). The administration of IL-17A in γδ T-cell KO mice could alter the plasma ALT level, but not in αβ T-cell KO mice (**Figure 3I**). However, *Rag1* mice that had adoptive transfer of IL-17A^Δ^ γδ T cells showed elevated levels of ALT to vehicle γδ T cells, suggesting that the protective role of γδ T cells in liver injury was IL-17A-dependent (**Figure 3J**). Thus, these data together suggest a major protective role of γδ T cells expressing IL-17A in sepsis-induced liver injury in CLP mice.

### Vγ4^+^ but not Vγ6^+^ γδ T cells were the major protectors in sepsis-induced liver injury

**Figure 4:**
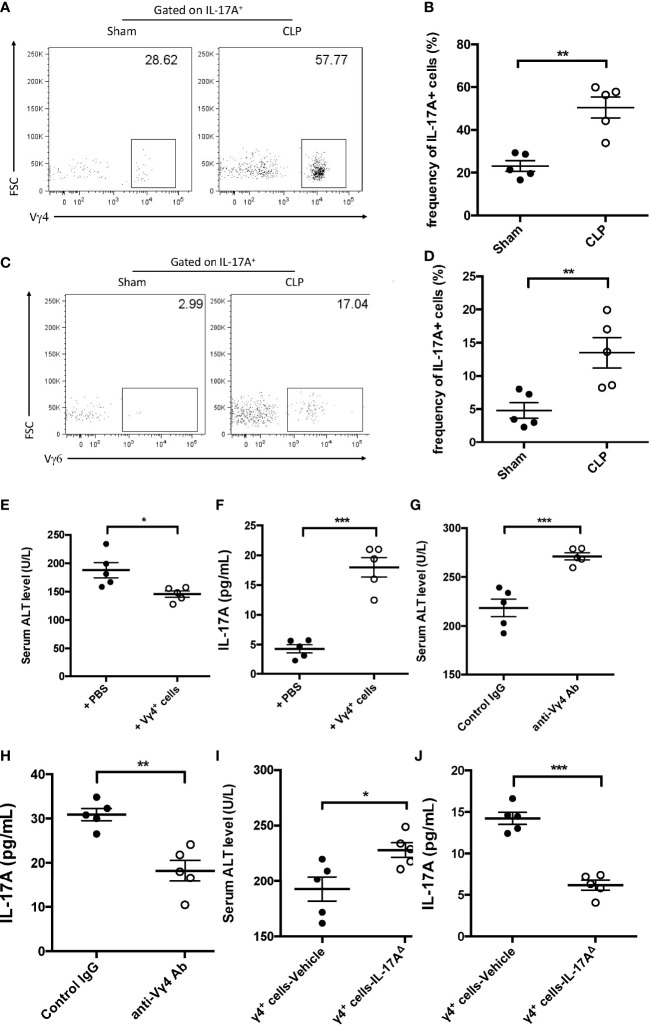
Function of Vγ4^+^ cells in cecum ligation and puncture (CLP)-induced liver injury. **(A, C)** Representative fluorescence-activated cell sorting (FACS) plots of Vγ4^+^ **(A)** and Vγ6^+^ **(C)** cells among the IL-17A^+^ cells between sham and CLP mice. **(B, D)** Statistics of Vγ4^+^ **(B)** and Vγ6^+^ **(D)** cells in (Cheng et al., 2017). *Rag1* mice were adoptive transferred with Vγ4^+^ cells and then subjected to CLP. **(E)** Serum levels of alanine transaminase (ALT) measured as described. **(F)** Hepatic levels of interleukin 17A (IL-17A) determined using ELISA. C57BL/6 mice were treated with control immunoglobulin G (IgG) or the anti-Vγ4 antibody through intraperitoneal injection and then subjected to CLP. **(G)** Serum levels of ALT measured as described. **(H)** Hepatic levels of IL-17A determined using ELISA. *Rag1* mice were adoptive transferred with BL-vehicle- or BL-IL-17A^Δ^-transfected Vg4^+^ cells and then subjected to CLP. **(I)** Serum levels of ALT measured as described. **(J)** Hepatic levels of IL-17A determined using ELISA. Data are from one intact experiment and representative of three independent experiments (five mice per group). *Error bars* denote the mean ± SEM. Statistical analysis was performed using Student’s *t*-test. *n.s.*, not significant. *p < 0.05, **p < 0.01, ***p < 0.001.

IL-17A-producing γδ T cells are mainly composed of two subsets: Vγ4 and Vγ6. These two subtypes of γδ T cells have been reported in many disease models and have presented distinguished functions (Ribot et al., 2009; Wu et al., 2014). Thus, the subsets of IL-17A-producing hepatic γδ T cells in mice subjected to CLP were further investigated. The FACS data showed that more IL-17A^+^ γδ T cells expressed the Vγ4 chain (**Figures 4A, B**) compared to the Vγ6 chain (**Figures 4C, D**). Adoptive transfer of Vγ4^+^ γδ T cells into *Rag1* mice could suppress the plasma ALT and elevate the hepatic IL-17A levels post-CLP compared to control animals (**Figures 4E, F**). Subsequently, Vg4^+^ γδ T cells were abolished in C57BL/6 mice using an anti-Vγ4 depletion antibody and the mice subjected to CLP. After 12 h, these mice presented altered liver IL-17A levels and severe liver injury (**Figures 4G, H**). Coordinately, *Rag1* mice transferred with IL-17A^Δ^ Vγ4^+^ γδ T cells had decreased levels of hepatic IL-17A and increased plasma ALT compared to vehicle Vγ4^+^ γδ T cells (**Figures 4I, J**). Therefore, these results determined a critical role of Vγ4^+^ γδ T cells in protecting against sepsis-induced liver injury through the secretion of IL-17A.

### Protective γδ T cells migrated into the liver after cecum ligation and puncture

**Figure 5:**
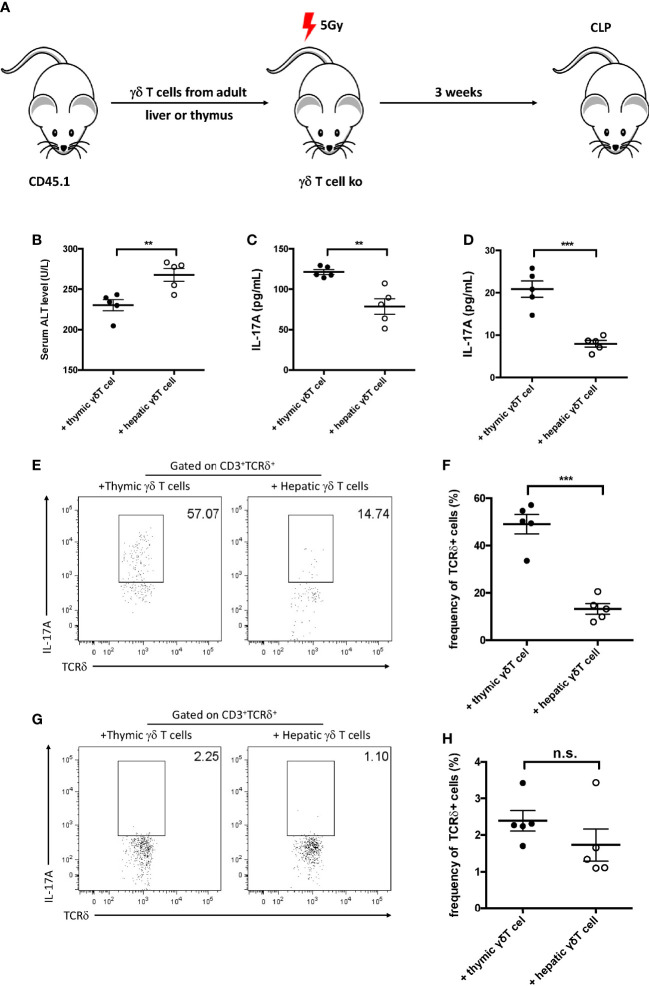
Gamma delta (γδ) T cells migrated into the liver after cecum ligation and puncture (CLP). **(A)** Schematic showing the experiment design. Gamma delta (γδ) T cells (1 × 10^5^) sorted from the liver or thymus of CD45.1 mice were intravenously transferred to 5 Gy-irradiated γδ T-cell knockout (KO) mice. Recipients were subjected to CLP 3 weeks later. **(B)** Serum levels of alanine transaminase (ALT) measured as described. **(C, D)** The plasma and hepatic levels of interleukin 17A (IL-17A) were determined using ELISA. **(E, G)** Representative fluorescence-activated cell sorting (FACS) plots of IL-17A^+^ cells among the TCRδ^+^ cells in the liver **(E)** and the spleen **(G)**. **(F, H)** Statistics of IL-17A^+^ cells in (Cheng et al., 2017). Data are from one intact experiment and representative of three independent experiments (five mice per group). *Error bars* denote the mean ± SEM. Statistical analysis was performed using Student’s *t*-test. **p < 0.01, ***p < 0.001.

As the development of γδ T cells originates from both the thymus and the liver, the origin of protective IL-17^+^ γδ T cells was subsequently explored in sepsis-induced liver injury. The γδ T cells from the liver and thymus were isolated and were intravenously injected into sublethally irradiated γδ T-cell KO recipients to determine their origin in the liver of septic mice (**Figure 5A**). The plasma ALT level was measured 12 h post-CLP and was found to significantly decrease in thymic γδ T-cell transfer recipients (**Figure 5B**). In addition, the hepatic and serum levels of IL-17A were both higher in thymic γδ T-cell transfer recipients compared to liver γδ T-cell transfer recipients (**Figures 5C, D**). FACS analysis showed that the proportion of hepatic IL-17A^+^ γδ T cells was much higher in mice that received thymic γδ T cells compared to those that received liver γδ T cells (**Figures 5E, F**). In contrast, the levels of splenic IL-17A^+^ γδ T cells in mice transferred with thymic γδ T cells and liver γδ T cells were comparable (**Figures 5G, H**). These results suggest that the thymic γδ T cells, but not the liver γδ T cells, played the protective role, and these cells migrated into the liver to protect against sepsis-induced liver injury.

### Infiltration of IL-17A^+^ γδ T cells into the liver was CCR6-dependent

**Figure 6:**
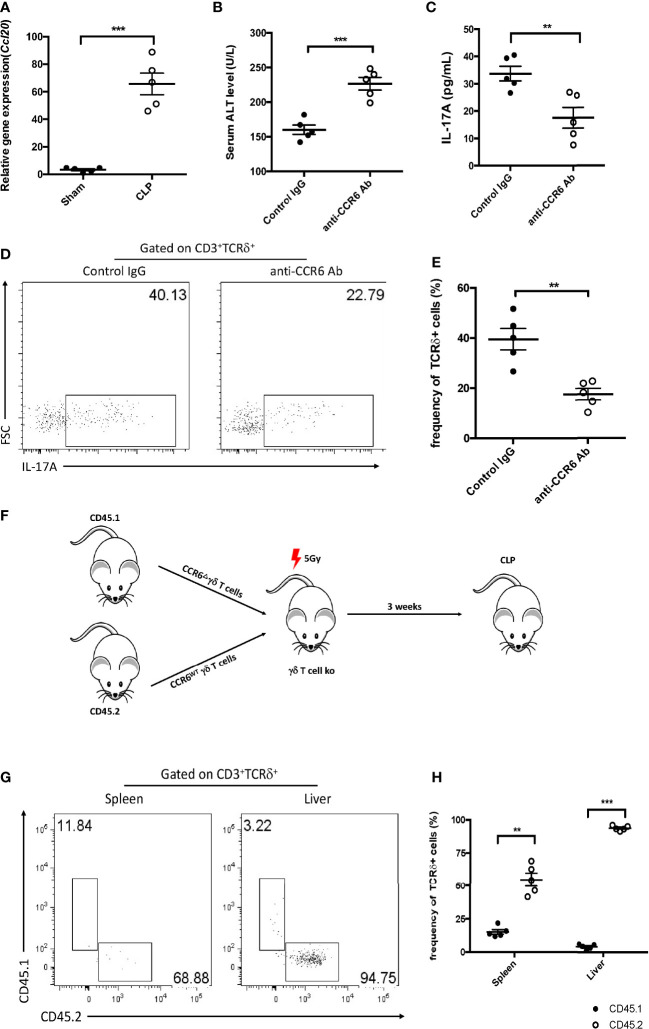
Function of CCR6 in gamma delta (γδ) T-cell migration into the liver. **(A)** Transcriptional levels of *Ccl20* in the liver between sham and cecum ligation and puncture (CLP) mice as determined by quantitative PCR (qPCR). C57BL/6 mice were treated with the control immunoglobulin G (IgG) or the anti-CCR6 antibody through intraperitoneal injection and then subjected to CLP. **(B)** Serum levels of alanine transaminase (ALT) measured as described. **(C)** Hepatic levels of interleukin 17A (IL-17A) determined using ELISA. **(D)** Representative fluorescence-activated cell sorting (FACS) plots of IL-17A^+^ cells among the TCRδ^+^ cells in the liver. **(E)** Statistics of IL-17A^+^ cells in (Cheng et al., 2017). **(F)** Schematic showing the experiment design. CD45.1^+^ γδ T cells were transfected with BL-CCR6^Δ^, while CD45.2^+^ γδ T cells were transfected with BL-vehicle; subsequently, these two populations were mixed at a ratio of 1:1 and adoptive transferred into 5 Gy-irradiated γδ T-cell knockout (KO) mice as recipients, which were subjected to CLP 3 weeks later. **(G)** Representative FACS plots of CD45.1^+^ and CD45.2^+^ cells from the spleen and liver of recipients. **(H)** Statistics of CD45.1^+^ and CD45.2^+^ cells in (Cheng et al., 2017). Data are from one intact experiment and representative of three independent experiments (five mice per group). *Error bars* denote the mean ± SEM. Statistical analysis was performed using Student’s *t*-test. *n.s.*, not significant. **p < 0.01, ***p < 0.001.

As IL-17A^+^ γδ T cells were recruited into the liver post-CLP, the manner of infiltration during liver injury was investigated next. The accumulation of γδ T cells in the liver in CCL4-induced hepatitis has been reported to be CCR6-dependent (Hammerich et al., 2014). We therefore examined whether this approach is also functional in sepsis-induced liver injury. Mice that had the CLP procedure showed significantly higher hepatic gene expression level of *Ccl20* compared to the controls (**Figure 6A**). To elucidate the functional relevance of CCR6 in sepsis-induced liver injury, CLP mice were treated with an anti-CCR6 blocking antibody, which could suppress the CCL20–CCR6 axis pathway. Compared to mice injected the rabbit IgG, those given the anti-CCR6 antibody showed elevated plasma ALT and decreased hepatic IL-17A levels (**Figures 6B, C**). Moreover, there was a significant decrease in the fraction of IL-17A^+^ γδ T cells among hepatic lymphocytes (**Figures 6D, E**). The expression of CCR6 was knocked out using the RNAi system, and then CD45.1^+^CCR6^Δ^ and CD45.2^+^CCR6^WT^ γδ T cells were mixed in a 1:1 ratio and injected intravenously into sublethally irradiated γδ T-cell KO mice (**Figure 6F**). At 12 h post-CLP, an overwhelming number of CD45.2 (i.e., CCR6^WT^) γδ T cells was found, but only a few CD45.1^+^ (i.e., CCR6^Δ^) γδ T cells in the liver (**Figures 6G, H**). Consistently, although only a few cells were detected in the spleen, the frequency of CD45.2^+^ γδ T cells was much higher than that of CD45.1^+^ γδ T cells (**Figures 6G, H**). These data together indicate that γδ T cells migrate into the liver during sepsis through a CCR6-dependent manner.

### Hepatic IL-17A^+^ γδ T cells were regulated by the microbiota in sepsis-induced liver injury

**Figure 7:**
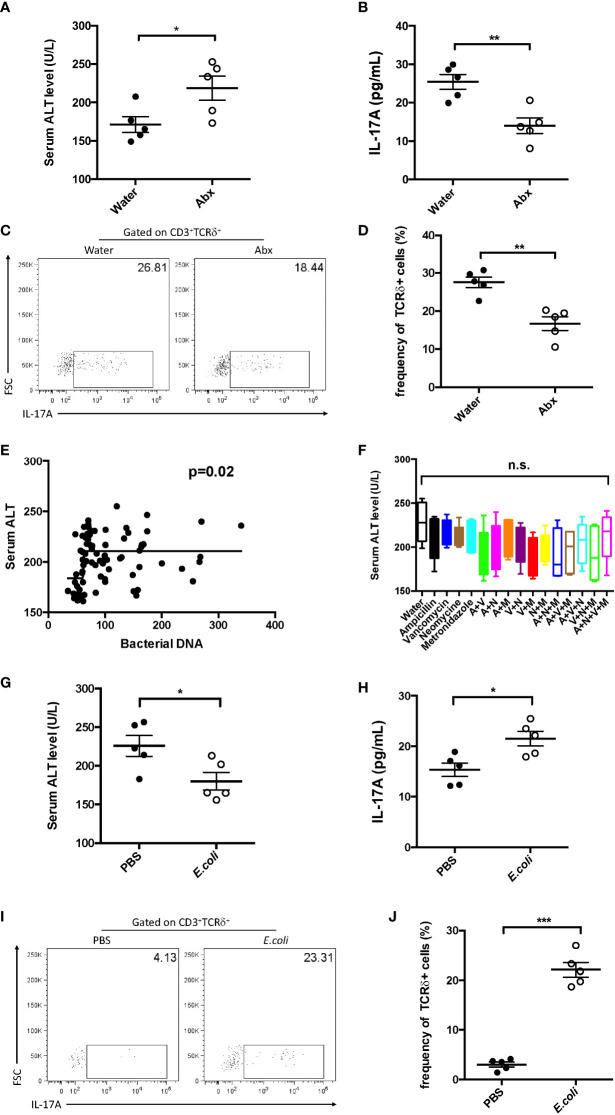
Effect of commensal microbes on cecum ligation and puncture (CLP)-induced liver injury. C57BL/6 mice were fed antibiotics for 5 days to deplete the gut microbiota, received fecal suspension for 3 days, and then subjected to CLP. **(A)** Serum levels of alanine transaminase (ALT) measured as described. **(B)** Hepatic levels of interleukin 17A (IL-17A) determined using ELISA. **(C)** Representative fluorescence-activated cell sorting (FACS) plots of IL-17A^+^ cells among TCRδ^+^ cells. **(D)** Statistics of IL-17A^+^ cells in (Cheng et al., 2017). **(E)** Pearson’s correlation curves between the serum ALT levels and bacterial DNA load. **(F)** C57BL/6 mice were fed water containing the following antibiotics: ampicillin (*A*), vancomycin (*V*), neomycin (*N*), and metronidazole (*M*). Pseudo-germ-free mice received fecal suspension for 3 days and then subjected to CLP. The serum levels of ALT were measured as described. Pseudo-germ-free mice intragastrically received 10^8^ *Escherichia coli* colony-forming units (CFUs) and then subjected to CLP. **(G)** Serum levels of ALT measured as described. **(H)** Hepatic levels of IL-17A determined using ELISA. **(I)** Representative FACS plots of IL-17A^+^ cells among TCRδ^+^ cells. **(J)** Statistics of IL-17A^+^ cells in (Cheng et al., 2017). Data are from one intact experiment and representative of three independent experiments (five mice per group). *Error bars* denote the mean ± SEM. Statistical analysis was performed using Pearson’s correlation or Student’s *t*-test. *p < 0.05, **p < 0.01, ***p < 0.001.

The gut microbiota has been considered as an important mediator of liver disease; thus, their function in hepatic IL-17A^+^ during sepsis was further investigated. Firstly, sepsis-induced liver injury in the absence of microbiota was analyzed. The absence of the microbiota led to increased plasma ALT and decreased hepatic IL-17A levels (**Figures 7A, B**). In addition, the frequency of IL-17A^+^ γδ T cells was much lower in pseudo-GF mice (**Figures 7C, D**).

To further investigate the function of bacterial species in sepsis-induced liver injury, mice were treated using different combinations of antibiotics (ampicillin, vancomycin, neomycin, and metronidazole). Subsequently, these mice were subjected to CLP. However, there was almost no difference among the antibiotic combinations (**Figure 7F**). In contrast, the serum level of ALT was positively correlated with the global microbial DNA load (**Figure 7E**). These results suggest that the global bacterial load has more significance in liver injury during sepsis.

Similar to the transfer of feces, the transfer of *Escherichia coli* alone could suppress the plasma ALT (**Figure 7G**) and recover the level of hepatic IL-17A (**Figure 7H**). Furthermore, in single *E. coli*-transferred mice, the fraction of hepatic IL-17A^+^ γδ T cells was strongly elevated (**Figures 7I, J**). These results indicate that the global microbiota, regardless of species, is crucial in sepsis-induced liver injury.

## Discussion

This study first clarified the role of IL-17A-producing γδ T cells in sepsis-induced liver injury, in which the Vγ4^+^ γδ T cell subtype played a protective role against liver injury during sepsis through the secretion of IL-17A. These cells originate in the thymus and migrate into the liver dependent on CCR6, and gut commensal microbes regulate this subset of γδ T cells.

Sepsis triggers systemic uncontrolled immune responses and could lead to organ dysfunction or even death. Over the last decade, research work on sepsis has demonstrated the protective role of γδ T cells (Flierl et al., 2008) and IL-17A (Ogiku et al., 2012), but the pathophysiology of sepsis-induced liver injury has not been clearly elucidated. Studies have shown that γδ T cells are the major source of IL-17A in sepsis (Kasten et al., 2010; Xu et al., 2010), indicating a potential correlation between IL-17A-producing γδ T cells and sepsis-induced liver injury. Consistent with previous findings, our data revealed that, aside from Th17 cells, IL-17A^+^ γδ T cells are a major source of IL-17A and that they play a crucial role in protecting against sepsis-induced liver injury.

Similar to αβ CD4^+^ T lymphocytes, subsets of γδ T cells may present distinct cytokine profiles, such as IFN-γ^+^ and IL-17A^+^ γδ T cells. Regarding IL-17A^+^ γδ T cells, it has been well established that IL-17A production is restricted to Vγ4^+^ and Vγ6^+^ γδ T cells among the total γδ T cells in mice (Bonneville et al., 2010; Rei et al., 2014). Consistent with these results, the data in this study revealed that Vγ4^+^ γδ T cells comprise the predominant subset among the IL-17A-producing cells in the liver of CLP mice. In line with these results, IL-17A^+^ γδ T cells comprise the major subset infiltrating into the liver with IL-17A production in different mouse models (Zhao et al., 2011; Hou et al., 2013). In addition, our data demonstrated that IL-17A predominated over IFN-γ from the γδ T cells in CLP mouse livers. Therefore, these data further supported that IL-17A^+^ Vγ4^+^ γδ T cells and not IFN-γ^+^ Vδ1^+^ γδ T cells predominated in CLP mouse liver injury.

The liver is the major target organ in sepsis. While this organ is metabolically active, it is immunologically quiescent. It has been shown that hepatocytes are capable of producing IL-7 to regulate the production of IL-17A in γδ T cells *in situ* during viral hepatitis infection (Hou et al., 2013). Hepatic γδ T cells originate from fetal γδ thymocytes and then migrate into the liver. Moreover, IL-17A^+^ γδ T cells could migrate into the liver from the periphery, similarly to other organs. It has been reported that IL-17^+^ γδ T cells in the skin (Gray et al., 2011; Naik et al., 2012), IL-17A^+^ γδ T cells residing in the lungs (Cheng et al., 2014), and IL-17A^+^ γδ T cells in colonic lamina propria (Lee et al., 2015) are all recruited from the periphery, but with different pathways to induce the production of IL-17A. In sepsis, protection was conferred by hepatic γδ T cells. Furthermore, our data revealed that the thymic and not the liver γδ T cells played the protective role in CLP, indicating that these cells were infiltrated from the periphery and are not resident cells, further clarifying the function of γδ T cells at different barrier sites with a tissue-specific approach.

The characteristics of liver injury include complex inflammatory responses between parenchymal and immune cells, no matter whether these immune cells are resident cells or were infiltrated. Recently, increased studies on chemokines and their receptors have revealed their functions in liver injury (Wasmuth et al., 2009; Karlmark et al., 2010). Our work demonstrated that the CCL20–CCR6 axis was functionally involved in sepsis-induced liver injury. Consistent with the findings in a mouse model, patients with cholestatic liver disease showed elevated CCR6 expression, which linked CCL20 to biliary epithelial cells (BECs) (Oo et al., 2012); however, these were considered as Th17-dominated. In this study, CCR6 was mainly expressed on hepatic γδ T cells with much higher levels than CD4 T cells in injured livers. Until now, little has been known about the function of CCR6^+^ γδ T cells *in vivo*, although these cells have also been detected in a colitis model (Mielke et al., 2013). Further functional studies of these subsets are needed.

Commensal microbes could drive the secretion of IL-17A in the gastrointestinal tract, which has been reported to maintain intestinal homeostasis (Ajuebor et al., 2008; Chen et al., 2013). The potential mechanism could be attributed to common components of the intestinal microbiota, such as lipid antigens, which could activate hepatic γδ T cells and induce IL-17A production. As indicated by our data, there was little difference between the antibiotic treatments, and *E. coli* alone could rescue the protection against sepsis in pseudo-GF mice. Reports have shown that IL-17A^+^ γδ T cells could protect against infection and promote inflammation in the liver (Chen et al., 2019). A study on hepatocellular carcinoma demonstrated that the absolute number of γδ T cells in the liver decreased in the absence of the gut microbiota, indicating a correlation between commensal microbiota and the number of hepatic γδ T cells (Schneider et al., 2022). These results were consistent with our findings regarding sepsis-induced liver injury. After the depletion of commensal microbes, the number of hepatic IL-17A^+^ γδ T cells decreased post-CLP (**Figures 7C, D**). It was also found that *E. coli* alone could restore hepatic IL-17A^+^ γδ T cells in CLP mice, which could be supported by the findings of Li et al. (2017). These findings all identified gut microbes as important regulators of hepatic IL-17A^+^ γδ T cells both in cancer and during infection.

In conclusion, this study clarified the function of Vγ4^+^ IL-17A-producing γδ T cells in sepsis-induced liver injury. These cells originate from the periphery and migrate into the liver in a CCR6-dependent manner, with the gut commensal microbes being the upstream regulators for this population.

## Data availability statement

The original contributions presented in the study are included in the article/supplementary material. Further inquiries can be directed to the corresponding author.

## Ethics statement

The animal study was reviewed and approved by the Ethics Committee for Animal Care and Use at Shanghai Pudong New Area People’s Hospital.

## Author contributions

JW and YS designed the study. JW, QZ, YH, ZT, and WS performed the experiments. JW, QZ, YH, ZT, WS, SC, LQ, WDS, and YS analyzed the data. JW and YS wrote the manuscript. All authors contributed to the article and approved the submitted version.
